# The knowledge, ability, and skills of primary health care providers in SEANERN countries: a multi-national cross-sectional study

**DOI:** 10.1186/s12913-019-4402-9

**Published:** 2019-08-27

**Authors:** Shizheng Du, Yuling Cao, Tong Zhou, Agus Setiawan, Myat Thandar, Virya Koy, Mohd Said Bin Nurumal, Hong Anh, Wipada Kunaviktikul, Yan Hu

**Affiliations:** 1 School of Nursing, Fudan University, 305 Fenglin Road, Shanghai, 200032 People’s Republic of China; 2 School of Nursing, Nanjing University of Chinese Medicine, Nanjing, China; 3 Faculty of Nursing, Universitas Indonesia Kampus UI, Depok, Jawa Barat Indonesia; 4 University of Nursing, Yangon, Myanmar; 5 Chief Nursing Officer/Nursing Focal Person in Cambodia for WHO-WPRO, Phnom Penh, Cambodia; 6 Kulliyyah of Nursing, International Islamic University, 25100 Kuantan, Pahang Malaysia; 7 School of Nursing, Phenikaa University, Hanoi, Vietnam; 8 Faculty of Nursing, Chiang Mai University, Chiang Mai, 50200 Thailand

**Keywords:** Primary health care, Knowledge, ability, and skills, Perceived capability, Cross-sectional survey, Multi-national study

## Abstract

**Background:**

Primary health care (PHC) is usually the initial point of contact for individuals seeking to access health care and providers of PHC play a crucial role in the healthcare model. However, few studies have assessed the knowledge, ability, and skills (capacity) of PHC providers in delivering care. This study aimed to identify the capacity of PHC providers in countries of the Southeast and East Asian Nursing Education and Research Network (SEANERN).

**Methods:**

A multi-national cross-sectional survey was performed among SEANERN countries. A 1–5 Likert scale was used to measure eight components of knowledge, ability, and skill of PHC providers. Descriptive statistics were employed, and radar charts were used to depict the levels of the three dimensions (knowledge, skill and ability) and eight components.

**Results:**

Totally, 606 valid questionnaires from PHC providers were returned from seven countries of SEANERN (China, Myanmar, Indonesia, Thailand, Vietnam, Cambodia, and Malaysia), with a responsive rate of 97.6% (606/621). For the three dimensions the ranges of total mean scores were distributed as follows: knowledge dimension: 2.78~3.11; skill dimension: 2.66~3.16; ability dimension: 2.67~3.06. Furthermore, radar charts revealed that the transition of PHC provider’s knowledge into skill and from skill into ability decreased gradually. Their competencies in four areas, including safe water and sanitation, nutritional promotion, endemic diseases prevention, and essential provision of drugs, were especially low.

**Conclusions:**

The general capacity perceived by PHC providers themselves seems relatively low and imbalanced. To address the problem, SEANERN, through the collaboration of the members, can facilitate the appropriate education and training of PHC providers by developing feasible, practical and culturally appropriate training plans.

**Electronic supplementary material:**

The online version of this article (10.1186/s12913-019-4402-9) contains supplementary material, which is available to authorized users.

## Background

The year of 2018 marked the 40th anniversary of the Alma-Ata Declaration on Primary Health Care (PHC) [1]. The Declaration on PHC was endorsed by all the countries and was considered a watershed in terms of the concepts and practices of public health as a scientific discipline. World Health Organization (WHO) advocated PHC as the key to attain the goal of “Health for All (HFA) in 2000” [1]. WHO defined PHC in Declaration of Alma-Ata as “essential health care based on practical, scientifically sound and socially acceptable methods and technology made universally accessible to individuals and families in the community through their full participation and at a cost that the community and country can afford to maintain at every stage of their development in the spirit of self-reliance and self-determination” [1].

Over the past 40 years, PHC provision has moved from thoughts and words to action and reality. Though the ambitious goals of HFA were not achieved in 2000, PHC, as a core value of WHO Constitution and Alma-Ata Declaration, is still crucial in current global health context [2], especially for developing countries [3]. The United Nations (UN) announced eight Millennium Development Goals (MDGs) by 2015 [4]. However, the achievements were uneven, and thus, the UN announced 17 Sustainable Development Goals (SDGs) and expected them to be achieved in 2030 [5]. These 17 goals should be taken seriously and actively implemented by all countries. Health and wellbeing is one of the SDGs and PHC is one way to achieve that goal. More specifically, Gillam stated that Alma-Ata Declaration is still relevant for effective health care systems, since the core principles of PHC are keys to moving towards health equity or universal health coverage under such a changing and challenging context [6]. In the World Health Report of 2008, WHO Director General Margaret Chan addressed that PHC deserved the greatest attention for policy makers and national governments [2]. Health systems which are oriented towards PHC are more likely to have better health outcomes and greater public satisfaction at lower costs and true access [7, 8]. PHC has usually been regarded as the initial point of contact for individuals wishing to access health care [1], especially for the vulnerable groups [6]**.** For example, about 75% of child and adolescent mental health problems were first treated by PHC providers, who were usually the first medical professionals consulted by children and their families [9]. Furthermore, the PHC links multiple sectors and disciplines, integrates a variety of elements of disease management, emphasizes prevention and early detection, and the maintenance of health. In fact, as the most affordable and accessible health service for residents, especially for those in remote areas, PHC has been long viewed as the “first element” as well as part of a “continuing health care process” [10].

Specifically, Alma-Ata Declaration has outlined eight essential components of PHC [1], including: (1) Health education on prevailing health problems and the methods of preventing and controlling them; (2) Nutritional promotion including food supply; (3) Supply of adequate safe water and sanitation; (4) Maternal and child health care; (5) Immunization against major infectious diseases; (6) Prevention and control of locally endemic diseases; (7) Appropriate treatment of common diseases and injuries; and (8) Provision of essential drugs. All these basic requirements are incorporated in the SDGs for 2030 from Goal 2 to Goal 4 [5].

Undoubtedly, providers of PHC play a crucial role in promoting, preventing, curing and rehabilitating residents. The appropriate delivery of PHC relies on physicians, nurses, midwives, community and ancillary workers, as well as traditional practitioners, suitably trained to work as a team and to respond to the expressed health needs of the community [1, 11]. However, due to the growing strain on resources and the shortage of PHC physicians and nurses in many countries, especially in developing countries [12, 13], policy-makers must consider new and creative opportunities to expand and enhance the provision of PHC [13].

From the updated population statistics of WHO [14], the Southeast and East Asian Nursing Education and Research Network (SEANERN) countries constitute almost 30% of the world’s population and 57.17% of the South-East Asia and Western Pacific Region. The SEANERN countries include 10 Association of Southeast Asian Nations (ASEAN) member states (i.e., Thailand, Singapore, Indonesia, Cambodia, Myanmar, Laos, Vietnam, Philippines, Malaysia, and Brunei) and 3 East Asian countries (i.e., China, Japan and Korea). Many SEANERN countries’ economies are considered as developing or transitioning and there are shortages of PHC providers in most of these countries [15, 16]. Importantly and specifically, a study within four of the SEANERN countries (Cambodia, Indonesia, Singapore and Vietnam) and India indicated that there was urgent need for more knowledge in terms of healthy ageing, life course approach, policies to support the access and quality of PHC for older people, and premature death can be reduced from chronic non-communicable diseases by means of applying effective PHC services [17]. A recent survey in China [18] showed that both physicians and patients of PHC rated “coordination” as the lowest score among the six indicators, including quality of care, equity, accessibility, continuity, coordination, and comprehensiveness. Therefore, it is an indispensable task to facilitate the coordination of care for PHC system reform in China. Thus, the capability of PHC providers of SEANERN countries are of far-reaching significance to provide accessible, qualified health care service for residents, especially for those in developing countries.

To date, there is sparse literature to investigate the knowledge, skills and abilities of PHC providers in this region. The purpose of this study was to characterize the knowledge, skills and abilities of PHC providers across SEANERN countries.

## Methods

### Study design

A descriptive multi-national cross-sectional study.

### Conceptual framework

The eight essential components of PHC outlined in the Alma-Ata Declaration were served as the framework. In addition, there were three dimensions of PHC providers’ capacity: knowledge, skill and ability [19]. Accordingly, the basic capacities of PHC providers consist of the knowledge of appropriate PHC service, relevant skills to provide PHC services, and the ability to provide appropriate PHC services.

### Sampling

By purposive sampling, PHC providers who implemented PHC services within the SEANERN countries were recruited, including community nurses, family doctors, staff in local clinics, and other health professionals. Inclusion criteria: (1) working on a PHC position in a SEANERN member country; (2) have worked in the two weeks prior to the survey; (3) volunteer to participate in the study.

### Instruments

A questionnaire (Additional file 1) was developed including demographic information and questions related to knowledge, skills and abilities.

The demographic information included participants’ country, age, gender, original education level, highest education level, job category, job title, years of working/clinical experience, and working years at current department.

For the capacity part, there were three dimensions (knowledge, skill & ability) on the eight components. Generally, for each of the three dimensions, all the eight items were self-rated by the participants on 5-point Likert scales, with 1 = whole-process guidance needed and 5 = expert.

Prior to the formal study, the instrument was presented to the expert panel from 13 SEANERN countries and preliminarily tested in China and then Thailand. The pilot study in China in April 2018 showed that the Cronbach’s α scores were 0.938, 0.963 and 0.961 for PHCs’ knowledge, skill and ability, respectively. According to the pilot study in Thailand in May 2018, Cronbach’s α values were 0.894,0.910 and 0.927, respectively.

To establish the face validity of the scale, a total of 13 experts from 13 SEANERN countries were assembled in Shanghai to review and assess the content of the English version of the questionnaire in April, 2018. All the experts agreed that the questionnaire conformed to the conceptual framework and the objective of the study.

Moreover, to ensure the successful application of the scale in non-English speaking countries, translations and linguistic validations of the questionnaire were performed by the experts of the country, with the procedure including forward translation, reconciliation, back translation, and discussion on cultural equity.

### Ethical statement and data collection

This study has been approved by Ethical Committee, School of Nursing, Fudan University (No. IRB# 2018-3-9). Additionally, the study was reviewed and approved by the institutional review board (IRB) of each institution involved in the study.

Between May and July 2018, the survey was implemented based on purposive sampling. The coordinators of each SEANERN country chose appropriate institutions in their own country, which covered as many types as possible of PHC institutions in the country. First, the approval of survey application was obtained from supervisors of each selected PHC center. Then the trained investigators went to the PHC center and invited the eligible PHC providers to assemble in a meeting room. The investigators introduced the aim of the survey, and then elaborated on the details of dimensions and items. After signing informed consent, PHC providers completed the questionnaire on their own. The coordinators of each country checked the completeness after the participants returned the questionnaire. The data were input into EpiData files. Finally, all the data from every country were sent to the principal investigator by Email for final combination and data analysis.

### Data analysis

Data were firstly analyzed by SPSS 23.0 (IBM Company, Chicago, IL, USA). Missing data were replaced with Mean Imputation. Descriptive statistics, such as frequency, percentage, mean ± standard deviation, were performed. It was also assessed whether assumptions of normal distribution (by Kolmogorov–Smirnov test) and homogeneity of variance (by Levene’s test) were met. Independent two samples t-test and one-way analysis of variance (ANOVA) were performed to analyze the differences in scores of knowledge, skill, and ability on eight components among samples with different demographic characteristics. Specifically, least significant difference (LSD) technique was used as post hoc analysis in ANOVA.

Additionally, radar charts in Microsoft Excel (version 2011) were also applied to visually depict the levels of three dimensions (knowledge, skill and ability) on the eight components. Three diagrams of radar charts were displayed. One diagram illustrated the scores of three dimensions on the eight components with all axes being scaled equally from 1 to 5 to show the general status of the capacity of PHC, while the other two diagrams displayed more detailed, specific information with all axes of the radar chart being scaled equally from 2.5 to 3.25, within which the mean value of all scores fluctuated, to vividly compare the scores of the three dimensions of the eight components.

## Results

### SEANERN countries involved in the study

Totally, 606 PHC providers agreed to participate in the survey and provided valid data, with a response rate of 97.6% (606/621), who were from seven countries of SEANERN (i.e., China, Myanmar, Indonesia, Thailand, Vietnam, Cambodia and Malaysia). For other countries of SEANERN, surveys had not been launched or completed by July 2018 due to the limited time for the review and approval by the IRB or specific financial reasons.

Table 1 displays the general information of the participants. Specifically, the mean age of the 606 participants were 36.9 ± 9.4, and females made up approximate 75% (460/606, 75.9%) in total. For their original education level, only seven (1.2%) held master degrees and none held doctoral degree. For their current education level, 28 (4.6%) had master degrees and only three (0.5%) had doctoral degrees. For the job category, nursing staff accounted for the largest proportion (56.1%), followed by other category (19.1%), medicine (12.5%), public health (10.1%), and pharmacy (2.2%). For the job title, the proportion of junior title was the largest (43.6%), followed by medium title (37.6%), senior title (14.2%), and others (4.6%), respectively. The average length of the working experience was (14.0 ± 8.9) years.

**Table 1:** The general information of the participants of PHC providers in the seven countries (*n* = 606)

Variables	China	Malaysia	Thailand	Vietnam	Indonesia	Myanmar	Cambodia	Total
Sample size	110	85	100	81	80	80	70	606
Age	36.5 ± 9.5	36.4 ± 11.5	40.5 ± 9.4	37.5 ± 8.7	36.9 ± 7.5	37.9 ± 11.1	30.4 ± 5.4	36.9 ± 9.4
Gender								
Male	18	7	19	14	15	12	61	146 (24.1%)
Female	91	78	81	67	66	68	9	460 (75.9%)
Original education level								
Diploma	52	22	35	71	32	10	57	279 (46.0%)
Bachelor	38	56	65	10	40	43	11	263 (43.4%)
Master	4	2	0	0	1	0	0	7 (1.2%)
Doctor	0	0	0	0	0	0	0	0 (0.0%)
Others	16	7	0	0	7	25	2	57 (9.4%)
Current highest education level								
Diploma	30	14	11	48	30	6	6	145 (23.9%)
Bachelor	73	67	76	31	41	47	65	400 (66.0%)
Master	4	5	15	0	2	2	0	28 (4.6%)
Doctor	0	0	1	0	0	1	1	3 (0.5%)
Others	3	1	0	0	9	17	0	30 (5.0%)
Job category								
Medicine	48	2	2	2	11	10	1	76 (12.5%)
Pharmacy	5	0	2	3	3	0	0	13 (2.2%)
Nursing	52	76	63	35	32	13	69	340 (56.1%)
Public health	4	7	10	5	3	32	0	61 (10.1%)
Others	1	0	24	35	31	25	0	116 (19.1%)
Job title^a^								
Junior	40	42	95	2	23	50	12	264 (43.6%)
Medium	47	11	9	44	34	29	54	228 (37.6%)
Senior	14	30	1	25	15	0	1	86 (14.2%)
Others	7	2	0	6	8	2	3	28 (4.6%)
Years of working experience	15.3 ± 9.8	13.9 ± 10.0	17.6 ± 9.4	13.3 ± 8.0	13.2 ± 7.8	12.0 ± 9.0	10.2 ± 5.4	14.0 ± 8.9
Years of clinical experience	15.0 ± 9.8	12.0 ± 9.1	12.4 ± 10.2	12.1 ± 8.0	12.5 ± 8.1	11.3 ± 8.8	9.7 ± 5.2	12.4 ± 8.9
Working years at current department	8.4 ± 8.0	5.1 ± 5.4	11.7 ± 7.5	12.7 ± 7.9	9.5 ± 8.5	7.0 ± 7.8	8.4 ± 4.7	9.6 ± 7.7

Abbreviation: *PHC* Primary Health Care

^a^Examples for different job titles:

*Junior:* e.g. medical assistant;

*Medium:* e.g. attending physician;

*Senior:* e.g., associate chief physician, chief physician

### Status of capacity PHC providers

Table 2 demonstrates all the scores of three dimensions on the eight components. For the three dimensions on eight components, the ranges of total mean scores were distributed as follows: knowledge dimension: 2.78~3.11; skill dimension: 2.66~3.16; ability dimension: 2.67~3.06. Notably, results of t-test analyses and ANOVA (including LSD post hoc analysis) revealed that there were some differences in capacity levels among patients with different demographic characteristics. For different genders, female PHC providers scored better than male counterparts in skill of maternal and child health care, and in ability of health education and maternal and child health care. For different job titles, PHC providers with medium title almost scored best in three domains on the all eight components. For different job categories, nurses scored higher than physicians in all three dimensions on certain components. Specific details are available in Note part of Table 2.

**Table 2:** Self-rated capacity levels of primary health care providers of the seven countries(n = 606)

Components of PHC	Dimensions		
	Knowledge^a^Mean ± SD	Skill ^b^ Mean ± SD	Ability^c^ Mean ± SD
Health education (C1)	3.11 ± 1.12	3.16 ± 1.15	3.06 ± 1.15
Nutritional promotion (C2)	2.89 ± 1.20	2.76 ± 1.17	2.71 ± 1.16
Supply of adequate safe water & sanitation (C3)	2.80 ± 1.32	2.66 ± 1.32	2.67 ± 1.29
Maternal & child health care (C4)	3.04 ± 1.27	2.93 ± 1.21	2.96 ± 1.25
Immunization (C5)	2.99 ± 1.31	2.95 ± 1.28	2.94 ± 1.26
Prevention & control of locally endemic diseases (C6)	2.78 ± 1.28	2.82 ± 1.26	2.75 ± 1.24
Appropriate treatment of common diseases & injuries (C7)	2.98 ± 1.29	2.95 ± 1.26	2.90 ± 1.26
Provision of essential drugs (C8)	2.90 ± 1.27	2.91 ± 1.18	2.85 ± 1.18

Note: Mean scores were rated on the scales which ranges from 1 to 5, with 1 being worst, and 5 being best

Based on independent two samples t-test and ANOVA (including LSD post hoc analysis), we found that:

^a^ For knowledge dimension, there were no differences in all the eight components between the two genders; there were differences in all the eight components (C1-C8) among different titles, and PCH providers with medium title all scored the best; there were differences in all the eight components (C1-C8) among different job categories, with pharmacists scoring the worst in seven components (C1-C7), and nurses scoring higher than physicians in five components (C2-C6)

^b^For skill dimension, female PHC providers scored higher than male in one component (C4); there were differences in all the eight components (C1-C8) among different titles, and PCH providers with medium title all scored the best; there were differences in all the eight components (C1-C8) among different job categories, with pharmacists scoring the worst in seven components (C1-C7), and nurses scoring higher than physicians in six components (C1-C6)

^c^For ability dimension, female PHC providers scored higher than male in two components (C1, C4); there were differences in seven components (C1-C2, C4-C8) among different titles, and PCH providers with medium title all scored the best; there were differences in all the eight components (C1-C8) among different job categories, with pharmacists scoring the worst in seven components (C1-C7), and nurses scoring higher than physicians in three components (C3-C5)

Abbreviations*: ANOVA* Analysis of Variance (ANOVA);

*LSD* Least Significant Difference;

*PHC* Primary Health Care

*SD* Standard Deviation

**Fig. 1:**
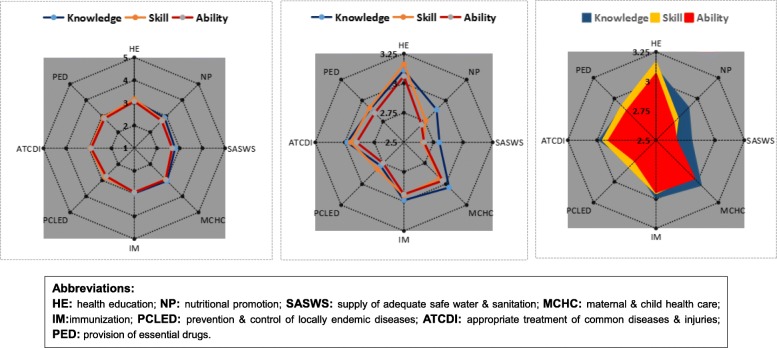
Radar chart of capacity levels of primary health care providers across the seven countries. Diagram I: Chart of overview; Diagram II: Chart of specific comparison; Diagram III: Chart of specific comparison (Fill)

Correspondingly, by means of radar chart, Fig. 1 also visually displays the total mean scores of the three dimensions on eight components of all the seven countries. As is depicted by Diagram I of Fig. 1, all the mean scores were plotted at around the point of 3 on all the eight axes. Moreover, Diagram I also revealed that the distribution graphs of knowledge, skill and ability were highly overlapped, indicating that the scores of three dimensions were relatively consistent with each other. In general, Diagram I showed that, on a 1–5 score scale, PHC providers were observed to have a low to moderate capacity in delivering PHC services.

More specifically, Diagram II and III show more details. From the two diagrams at a more micro level**,** it was observed that the knowledge dimension (in blue) occupied the largest area, followed by skill dimension (in yellow) and ability dimension (in red). This comparison showed there were some differences across the three dimensions of capacity, although their mean scores were around 3. Generally, the knowledge score of PHC providers was the highest, followed by skill score, and ability score was the lowest. Moreover, the scores on the eight components also varied. Diagram II visually demonstrates that, among the eight components of capacity, health education scored the highest in terms of knowledge, skill and ability dimensions (all above the score of 3). In comparison, four out of the eight components scored relatively low, including supply of adequate safe water and sanitation, nutritional promotion, prevention and control of locally endemic diseases, and provision of essential drugs.

## Discussion

### Principal findings

This is the first multi-national cross-sectional survey to quantitatively characterize the capacity of PHC providers in delivering health care services. The main findings indicate that the capacity of PHC providers was low to moderate in all three dimensions across the eight components. The overall score of the knowledge dimension was the highest, followed by the skill dimension, and the ability dimension was the lowest. For the eight components, health education scored the highest, while four components, including supply of adequate safe water and sanitation, nutritional promotion, prevention and control of locally endemic diseases, and provision of essential drugs, were observed to score relatively low.

### Gap of capacity requirement and status quo for PHC providers

The findings of this study revealed that PHC providers had a low to moderate perceived capacity in delivering PHC services, suggesting that there is a large gap between the requirements of PHC system and the actual performance of PHC providers. The result was consistent with conclusions of other relevant studies. As Gotovac et al. has specified [9], although the providers of PHC play an important role in assessment and management of the residents’ health problems, there is a great lack of adequate formal training for them. Specifically, a national study in Canada showed that there was a lack of confidence, skills, and adequate training in mental health care for PHC providers, and the providers themselves also recognize the gap and express their interest in training programs for building and strengthening their capacity if made available [20].

More specifically, at the macro level, all the seven countries involved in this study are developing countries. According to the classification of World Bank (2018) [21], the seven countries are categorized into two subgroups: China, Malaysia and Thailand are classified as upper middle income countries, and Vietnam, Indonesia, Myanmar, Cambodia are as lower middle income countries. In general, the under-developed economic status of these countries makes it difficult to realize PHC. One of the provisions of HFA was that 5 % of national gross national product should be devoted to health budget; however, most healthcare budget is allocated to large urban-based hospitals, such as secondary and tertiary health care centers, and PHC services appear to be underappreciated by the governments, resulting in insufficient financial funding and inadequate training for PHC providers [3, 22, 23].

The results also indicate that transitioning knowledge into skill and skill into ability should be the focus of any training for PHC providers. Of course knowledge is the foundation, but ability is destination. Thus, it is assumed that PHC providers would perform better if provided with adequate training on applying theoretical knowledge into practice.

This study also informs us that there is a pressing need for PHC providers to receive professional training in four areas, including supply of adequate safe water and sanitation, nutritional promotion, prevention and control of locally endemic diseases, and provision of essential drugs. Although the total level of PHC providers’ perceived capacity remains underdeveloped, the low scores in these four components signals to policy makers where the special attention is more urgent.

In addition, few of the PHC providers were found with high academic degree. Specifically, out of the 606 subjects, only 28 (4.6%) held master degrees and three (0.5%) held doctoral degrees, which may partly explain the relatively underdeveloped status for PHC providers’ knowledge, skill and ability. This survey illustrated that for the seven countries of SEANERN, the level of academic degree for PHC providers was generally low, which limits the delivery of high-quality PHC services to people. Higher Education program is therefore in urgent need for PHC providers.

### Next steps considerations

Obviously, the current status of PHC providers’ capacity cannot meet the requirement to realize the goal of SDGs by UN in 2030. As Xu et al. has specified recently [24], one of the factors accounting for this phenomenon lies in the failure of strategic emphasis on contemporary high-quality health education and professional training. Training programs are strongly recommended and should be carefully designed to address the low to moderate level of capacity of PHC providers. Specifically, training programs should incorporate methods for transforming theory into practice and specific content related to safe water and sanitation, nutrition, endemic disease prevention, and essential drugs.

SEANERN, established in 2013, is a network nursing school deans and senior faculty from 13 Southeast and East Asia countries who meet annually and are uniquely prepared and positioned to tackle the issue of PHC provider education and training [25]. More specifically, (1) They would develop a train-the-trainer model to deliver the high-quality training program; (2) Implement newly emerging training modalities such as participatory training, interactive case-based learning, high-fidelity simulation training, and web-based learning; (3) Tailor the training priorities to different levels of PHC personnel, e.g., entry level training to novices, enhanced training to backbone staff, and management training for administrators. PHC members who scored lower in this survey deserve special attention and targeted training programs can be designed and provided for them. (4) Establish a network for multinational cooperation within the already established SEANERN network to share successful experiences, and problem solving. It is hoped that, by these comprehensive countermeasures, feasible and practical training plan can be formulated for PHC providers in terms of programs’ purpose, contents, models of implementation, preparation of teaching staff, and improve their capacity for delivering PHC services.

### Implications

The findings may reflect the general status of PHC providers’ capacity in developing countries, which provide some evidence and meaningful implications to understand the underdeveloped PHC service level in nations with developing or transitioning economy. SEANERN, one of the international nursing organizations, is expected to play a positive, constructive role in improving PHC service level by facilitating multilateral coordination and program implementation.

### Limitations

Several limitations should be noted. Firstly, the principle of random sampling was not applied during the process for enrolling participants. To some extent, purposive sampling and relatively small sample size limit the representativeness of the population in each of the seven countries. Secondly, due to the limited time and financial shortage, the survey could be successfully implemented only within seven out of the 13 countries of SEANERN, which may reduce the internal validity of the findings. This can be considered as an obstacle to transferring the results and implications inside and outside of SEANERN. Thirdly, there are multiple stakeholders in PHC service and this study focused solely on the PHC providers with a self-rated scale. Lastly, this survey was based on a self-report questionnaire, which may introduce the reporting bias, and the findings based on participants’ perceptions should be interpreted with caution.

## Conclusions

With a multi-national survey, this study investigated the status of capacity of PHC providers in seven countries of SEANERN. This study implies that the capacity perceived by PHC providers themselves remains underdeveloped and there is much room for progress. In particular, the transforming capacity from knowledge into skill and from skill into ability is relatively poor, and their competencies in four areas, including safe water and sanitation supply, nutritional promotion, endemic diseases prevention, and essential drugs provision, are of great concern. To address the problem, SEANERN should be considered as a network that is well positioned to facilitate PHC training within the member countries, and feasible, practical training plans will be formulated for future international collaboration, especially for developing countries.

## Additional file

Additional file 1: Questionnaire (English version). (DOCX 23 kb)

## Data Availability

The datasets used and/or analyzed during the current study are available from the corresponding author Professor Yan HU on reasonable request.

## Abbreviations

ANOVA: Analysis of variance
ASEAN: Association of Southeast Asian Nations
HFA: Health for All
IRB: Institutional review board
LSD: Least Significant Difference
MDGs: Millennium Development Goals
PHC: Primary Health Care
SDGs: Sustainable Development Goals
SEANERN: Southeast and East Asian Nursing Education and Research Network
UN: United Nations
WHO: World Health Organization
