# Supplementary material for: The knowledge, ability, and skills of primary health care providers in SEANERN countries: a multi-national cross-sectional study
Source: BMC Health Serv Res. 2019 Aug 27;19:602. doi: 10.1186/s12913-019-4402-9 (PMC6712608; doi:10.1186/s12913-019-4402-9)
Supplement: Supplementary file 1 — Questionnaire (English version). (DOCX 23 kb) [file 12913_2019_4402_MOESM1_ESM.docx]

**Additional file 1**

**PHC Provider Questionnaire**

Country Number

**Demographic Data Form**

(**PHC provider version**)

**Instruction**: please mark the appropriate response (by “√”) or complete the information required.

| **1. What is your age? _______** | **2. What is your gender?**  a.  Male  b.  Female |
| --- | --- |
| **3. Your original education level**  a.  Diploma  b.  Bachelor  c.  Master  d.  Doctor  e.  other (specify) | **4. Your highest education level**  a.  Diploma  b.  Bachelor  c.  Master  d.  Doctor  e.  other (specify) |
| **5. Your original major**  a.  Medicine  b.  Pharmacy  c.  Nursing  d.  Public health  e.  other (specify) | **6. Your job title/position:**  a.  junior  b.  medium  c.  senior  d.  other (specify) |
| **7. Years of working experience:** ________  **Years of clinical experience:** ________ | |
| **8. Current work department:**  **Working years at current department:** ________ | |
| **9. Current work and frequency (you may choose up to 3, please tick ✓ and fill in)**   1. health education hours/week 2. nutrition promotion hours/week 3. environmental sanitation hours/week 4. family planning hours/week 5. maternal healthcare hours/week 6. child healthcare hours/week 7. vaccination hours/week 8. prevention and healthcare hours/week 9. medication hours/week 10. pharmacy hours/week 11. rehabilitation hours/week 12. Other (specify)______________ hours/week | |
| **10. Training and frequency having attended (multiple choices)**   1. written material times/year 2. audio material times/year 3. visual material times/year 4. workshop times/year 5. on-the-spot guidance times/year 6. online course times/year 7. short-term face-to-face course times/year 8. Other (specify)______________ times/year | |

**Primary Health Care Capacity Scale**

**(PHC provider version)**

Instruction: this scale is used to assess primary health care (PHC) capacity of PHC providers. Please read each item carefully and use score "1-5" to assess the level that fits your current situation. “NA” means “Not Applicable”, and “1” means “whole-process guidance needed”, 2 means “assistance needed”, 3 means “minimal support needed”, 4 means “proficient”, 5 means “expert”. There are no right or wrong answers, please answer as accurately as possible. Thank you!

| **I． Knowledge** | | | | | | | |
| --- | --- | --- | --- | --- | --- | --- | --- |
| **I have the required knowledge of** | | **Options** | | | | | |
|  |  | NA | 1 | 2 | 3 | 4 | 5 |
| a. health education on prevailing health problems, including methods to prevent and control them | |  |  |  |  |  |  |
| b. nutritional promotion, including food supply | |  |  |  |  |  |  |
| c. supply of adequate water and sanitation | |  |  |  |  |  |  |
| d. maternal and child health care | |  |  |  |  |  |  |
| e. immunization against major infectious diseases | |  |  |  |  |  |  |
| f. prevention and control of locally endemic diseases | |  |  |  |  |  |  |
| g. appropriate treatment of common diseases and injuries | |  |  |  |  |  |  |
| h. provision of essential drugs | |  |  |  |  |  |  |
| i. Other (please complete) | |  |  |  |  |  |  |
| j. In your opinion, which knowledge above is the **most required and least required** for your work? Please write the letter according to the order above (e.g., if you think immunization is the one you need the most, then write “e” in the brackets; if not in the list, please also write down in the brackets ) and give your reasons for it. | | | | | | | |
| the **most** required knowledge: ( )  Reasons: | the **least** required knowledge: ( )  Reasons: | | | | | | |
| k. In your opinion, which knowledge do you **need the most training & the least training** for your work? Please write the letter according to the order above (e.g., if you think immunization is the most lack of, and then write “e” in the brackets; if not in the list, please also write down in the brackets) and give your reasons for it. | | | | | | | |
| the knowledge need the **most** training: ( )  Reasons: | the knowledge need the **least** training: ( )  Reasons: | | | | | | |
| l. For the knowledge that you **need the most training**, what will help you most to fulfill it (please circle the one you choose)? And why?  Handouts Books Weblink Video Workshop Course Others, pls clarify( )  Reasons: | | | | | | | |
| **II． Skills** | | | | | | | |
| **I have the required skill for** | | **Options** | | | | | |
|  |  | NA | 1 | 2 | 3 | 4 | 5 |
| a. health education on prevailing health problems, including methods to prevent and control them | |  |  |  |  |  |  |
| b. nutritional promotion, including food supply | |  |  |  |  |  |  |
| c. supply of adequate water and sanitation | |  |  |  |  |  |  |
| d. maternal and child health care | |  |  |  |  |  |  |
| e. immunization against major infectious diseases | |  |  |  |  |  |  |
| f. prevention and control of locally endemic diseases | |  |  |  |  |  |  |
| g. appropriate treatment of common diseases and injuries | |  |  |  |  |  |  |
| h. provision of essential drugs | |  |  |  |  |  |  |
| i. Other (please complete) | |  |  |  |  |  |  |
| j. In your opinion, which skill above is the **most required and least required** for your work? Please write the letter according to the order above (e.g., if you think immunization is the one you need the most, then write “e” in the brackets; if not in the list, please also write down in the brackets ) and give your reasons for it. | | | | | | | |
| the **most** required skill: ( )  Reasons: | the **least** required skill: ( )  Reasons: | | | | | | |
| k. In your opinion, which skill do you **need the most training & the least training** for your work? Please write the letter according to the order above (e.g., if you think immunization is the most lack of, and then write “e” in the brackets; if not in the list, please also write down in the brackets) and give your reasons for it. | | | | | | | |
| the **most** need training skill: ( )  Reasons: | the **least** need training skill: ( )  Reasons: | | | | | | |
| l. For the skill that you **need the most training**, what will help you most to fulfill it (please circle the one you choose)? And why?  Handouts Books Weblink Video Workshop Course Others, pls clarify( )  Reasons: | | | | | | | |
| **III． Abilities** | | | | | | | |
| **I have the required ability for** | | **Options** | | | | | |
|  |  | NA | 1 | 2 | 3 | 4 | 5 |
| a. health education on prevailing health problems, including methods to prevent and control them | |  |  |  |  |  |  |
| b. nutritional promotion, including food supply | |  |  |  |  |  |  |
| c. supply of adequate water and sanitation | |  |  |  |  |  |  |
| d. maternal and child health care | |  |  |  |  |  |  |
| e. immunization against major infectious diseases | |  |  |  |  |  |  |
| f. prevention and control of locally endemic diseases | |  |  |  |  |  |  |
| g. appropriate treatment of common diseases and injuries | |  |  |  |  |  |  |
| h. provision of essential drugs | |  |  |  |  |  |  |
| i. Other (please complete) | |  |  |  |  |  |  |
| j. In your opinion, which ability above is the **most required and least required** for your work? Please write the letter according to the order above (e.g., if you think immunization is the one you need the most, then write “e” in the brackets; if not in the list, please also write down in the brackets ) and give your reasons for it. | | | | | | | |
| the **most** required ability: ( )  Reasons: | the **least** required ability: ( )  Reasons: | | | | | | |
| k. In your opinion, which ability do you **need the most training & the least training** for your work? Please write the letter according to the order above (e.g., if you think immunization is the most lack of, and then write “e” in the brackets; if not in the list, please also write down in the brackets) and give your reasons for it. | | | | | | | |
| the **most** need training ability: ( )  Reasons: | the **least** need training ability: ( )  Reasons: | | | | | | |
| l. For the ability that you **need the most training**, what will help you most to fulfill it (please circle the one you choose)? And why?  Handouts Books Weblink Video Workshop Course Others, pls clarify( )  Reasons: | | | | | | | |
| **Further comments:** | | | | | | | |

**This is the end of the questionnaire. Please make sure that you have filled all the items. Thank you!**
